# Cyantraniliprole and Thiamethoxam Exposure Changes Expression of Transcripts Associated with Small Non-Coding RNA Processing in the Colorado Potato Beetle

**DOI:** 10.3390/insects15030147

**Published:** 2024-02-22

**Authors:** Pierre Bastarache, Kenan Timani, Mariem Ben Youssef, Enock Omakele, Jess L. Vickruck, Pier Jr. Morin

**Affiliations:** 1 Department of Chemistry and Biochemistry, Universite de Moncton, 18 Antonine-Maillet Avenue, Moncton, NB E1A 3E9, Canada; epb6696@umoncton.ca (P.B.); ekt7790@umoncton.ca (K.T.); emb0892@umoncton.ca (M.B.Y.); eeo8493@umoncton.ca (E.O.); 2 Fredericton Research and Development Centre, Agriculture and Agri-Food Canada, 95 Innovation Road, Fredericton, NB E3B 4Z7, Canada; jess.vickruck@agr.gc.ca

**Keywords:** Colorado potato beetles, cyantraniliprole, microRNAs, non-coding RNAs, thiamethoxam, RNAi

## Abstract

**Simple Summary:**

The Colorado potato beetle is known for its ability to cause significant damage to potato crops worldwide. Unfortunately, this insect is also capable of developing substantial resistance against different compounds deployed to regulate its spread. A better understanding of the molecular basis underlying this resistance is key in order to develop novel strategies aimed at this potato pest. The goal of the current study was, thus, to characterize the expression of transcripts with likely relevance for insecticide resistance in Colorado potato beetles treated with two compounds of interest. Changes were observed for several transcripts in response to either low- or high-dose exposure, as well as for different time durations. The modulation of one transcript using RNAi was also associated with the increased susceptibility of insects treated with the insecticide cyantraniliprole. Overall, this information highlights potentially relevant targets for insecticide resistance in the Colorado potato beetle and reveals a transcript of interest for investigating further within the context of cyantraniliprole response in this pest.

**Abstract:**

The Colorado potato beetle (*Leptinotarsa decemlineata* (Say)) can cause extensive damage to agricultural crops worldwide and is a significant insect pest. This insect is notorious for its ability to evade various strategies deployed to control its spread and is known for its relative ease in developing resistance against different insecticides. Various molecular levers are leveraged by *L. decemlineata* for this resistance to occur, and a complete picture of the genes involved in this process is lacking. While small non-coding RNAs, including miRNAs, are differentially expressed in insects exposed to insecticides, levels of transcript coding for proteins underlying their synthesis remain to be characterized fully. The overarching objective of this work aims to fill that gap by assessing the expression of such targets in *L. decemlineata* exposed to cyantraniliprole and thiamethoxam. The expression status of *Ago1*, *Ago2*, *Ago3*, *Dcr2a*, *Dcr2b*, *Expo-5*, *Siwi-1* and *Siwi-2* transcripts were quantified via qRT-PCR in adult *L. decemlineata* treated with low and high doses of these compounds for different lengths of time. Variation in *Ago1* and *Dcr2b* expression was notably observed in *L. decemlineata* exposed to cyantraniliprole, while thiamethoxam exposure was associated with the modulation of *Dcr2a* and *Siwi-1* transcript levels. The down-regulation of *Ago1* expression in *L. decemlineata* using dsRNA, followed by cyantraniliprole treatment, was associated with a reduction in the survival of insects with reduced *Ago1* transcript expression. Overall, this work presents the insecticide-mediated modulation of transcripts associated with small non-coding RNA processing and showcases *Ago1* as a target to further investigate its relevance in cyantraniliprole response.

## 1. Introduction

The Colorado potato beetle, *Leptinotarsa decemlineata* (Say), can cause significant damage to the agricultural sector in various geographic locations worldwide and has been monitored closely, notably because of its rapid spread [1]. This insect is a notorious pest and can lead to substantial damage to potato crops, as exemplified by evidence that 40 cm^2^ of potato plant foliage can be consumed daily by *L. decemlineata* larvae [2]. Multiple methods have been deployed to control this insect, including the use of various chemical-based approaches with differing levels of efficacy. This has prompted the exploration of alternative avenues to regulate this pest, as well as the development of a better characterization of the molecular levers underlying this resistance [3,4]. Decreased susceptibility against various compounds, including chemicals associated with the neonicotinoids or the diamide class of insecticides, has been reported to various degrees in *L. decemlineata* [5,6,7,8]. Investigation into the molecular changes associated with responses against insecticides or toxins in *L. decemlineata* has highlighted multiple factors warranting closer characterization, including enzymes such as cytochrome P450 monoxygenases, glutathione s-transferases, glutathione synthetase and UDP-glycosyltransferases [9,10,11], as well as other targets of interest, including ATP-binding cassette transporter [12]. Nevertheless, while these studies identify novel genes to target via different approaches in order to mitigate the agricultural impact associated with *L. decemlineata*, the molecular changes associated with insecticide response in this pest have not been characterized completely.

Reports showing the modulation of small non-coding RNAs in response to insects treated with various insecticides have added another layer of molecular complexity in the process of deciphering insecticide response. The modulation of microRNAs (miRNAs), short non-coding RNAs that impact the post-transcriptional expression of a gene, has been demonstrated in various insects and has been linked with insecticide response. For example, one study showed the differential expression of seven miRNAs in the European honey bee, *Apis mellifera*, that had been exposed to a field-representative dose of thiamethoxam for a period of ten days [13]. Similarly, the treatment of Asian spongy moth *Lymantria dispar* larvae with cyantraniliprole has also revealed the differential expression of 23 miRNAs, further supporting the link between these small non-coding RNAs and insecticide responses [14]. Interestingly, the modulation of miRNAs in response to insecticide treatments has also been reported in *L. decemlineata*. The differential expression of 14 miRNAs was reported in adult *L. decemlineata* exposed to spinosad [15], and the modulation of 33 miRNAs was observed in adult *L. decemlineata* treated with imidacloprid [16]. Besides miRNAs, the short non-coding RNAs known as the PIWI-interacting RNAs (piRNAs), notably involved in germline maintenance, are also emerging as small molecules that warrant additional investigation for their potential involvement in insecticide resistance, as exemplified by studies that showcased the modulation of two piRNAs in response to pyrethroids in the mosquito *Culex pipiens pallens* [17,18]. It is also relevant to point out the observed differential expression of *Dcr-like* and *Ago2* transcripts—the latter being a key player in small non-coding RNA functions—in honey bees fed with clothianidin [19]. While there is mounting evidence that small non-coding RNAs are frequently modulated in insects exposed to insecticides, including *L. decemlineata*, the information is sparse with respect to the activity of the pathways underlying their biogenesis or function.

Overall, this study aimed to assess levels of select transcripts coding for proteins with relevance to small non-coding RNA processing. The data collected present fluctuating expressions of several transcripts of interest for non-coding RNA processing in an agricultural pest exposed to the diamide cyantraniliprole and the neonicotinoid thiamethoxam, as well as explore the impact of varying the levels of Ago1 in the context of insecticide response.

## 2. Materials and Methods

### 2.1. Insects

Adult *L. decemlineata* were sourced from the Fredericton Research and Development Centre (Fredericton, NB, Canada) in November 2022 and February 2023. Adults were transported to Université de Moncton in plastic containers in which potato (*Solanum tuberosum* var. Shepody) leaves had been deposited. Insects were initially held for at least 72 h in an insect mesh cage placed in an incubator (Thermo Fisher Scientific, Waltham, MA, USA). Temperature was set at 25 °C, and insects were maintained under 16L:8D cycles. Potato plants were made available to insects while in the incubator.

For cyantraniliprole (#32372, Sigma-Aldrich, St. Louis, MO, USA) exposure, a solution of 0.05 µg/µL of cyantraniliprole was prepared in acetone. A volume of 1 µL was sampled from this solution, equivalent to a dose of 0.05 µg, and subsequently deposited on the abdomens of 35 insects. In parallel, 35 control insects were treated with 1 µL of acetone. Approximately half of the insects from both the control and the treated groups were rapidly placed in liquid nitrogen 4 h after the initial applications and stored at −80 °C. The remaining insects were kept in the incubator longer and were sampled using the same approach 24 h after the initial applications. Cyantraniliprole exposure was also performed in parallel using groups of 20 insects treated with 0.01 µg, 0.05 µg, 0.1 µg, 0.5 µg, 1 µg or 5 µg of cyantraniliprole. This bioassay was primarily conducted to evaluate the toxicity of this compound in adult *L. decemlineata* and identify the doses of interest. A marked increase was observed in activity impairment and insect lethality—as evaluated using dish agitation and assessment of an insect’s capability to right itself [6,20]—between 0.05 µg and 0.1 µg one day following treatment (Table S1A). As a result, an initial lower dose of 0.05 µg was used at the study onset. In addition, subsequent cyantraniliprole treatment at a higher dose was also conducted to assess changes between low- and high-dose treatments. A total of 40 insects were exposed to a volume of 1 µL of a cyantraniliprole solution at a concentration of 0.25 µg/µL. This was equivalent to a final dose of 0.25 µg, or five times the amount of cyantraniliprole applied previously. As above, 1 µL of acetone was applied in parallel to insects used as controls. Insects were placed in the incubator following treatments for a duration of 4 or 24 h and collected in liquid nitrogen as above.

For thiamethoxam (#37924, Sigma-Aldrich) treatments, a similar approach was used. Abdominal application of 1 µL of a 0.1 µg/µL solution of thiamethoxam, prepared in acetone, was conducted on 35 insects. This was equivalent to a dose of 0.1 µg. A volume of 1 µL of acetone was also topically applied to 35 control insects. Approximately half of the exposed insects in both groups were sampled in liquid nitrogen 4 h post-treatment, while the remaining insects were sampled similarly 24 h after the initial topical applications. Preliminary testing was also conducted, as above with cyantraniliprole, on groups of 20 insects exposed to thiamethoxam doses equivalent to 0.01 µg, 0.05 µg, 0.1 µg, 0.5 µg, 1.0 µg and 5.0 µg. An increase in activity impairment and insect lethality was observed between 0.1 µg and 0.5 µg one day after thiamethoxam exposure, and the former dose was thus used for the initial treatments (Table S1B). This aligned with LD_50_ values previously reported for thiamethoxam in *L. decemlineata* [21]. Treatments using higher doses of thiamethoxam corresponding to five times the low dose, or 0.5 µg, were conducted as above on a group of 40 insects with durations of 4 or 24 h. Insect collection and storage were performed before total RNA isolation, as described above.

### 2.2. RNA Isolation

Isolation of total RNA was conducted with two adult *L. decemlineata* per replicate using the TRIzol reagent and following the provided protocol (Thermo Fisher Scientific). Concentration of total RNA was subsequently measured with a NanoDrop One microvolume spectrophotometer (Thermo Fisher Scientific). Total RNA was placed at −80 °C prior to conducting synthesis of cDNA.

### 2.3. Synthesis of cDNA

A total of 1 μg total RNA was sampled to undertake cDNA synthesis, as described previously [22]. Briefly, RNA was mixed with oligo dT, dNTPs and RNase-free water to 12 µL. Incubation at 65 °C for 5 min was conducted next, followed by addition of 5X First-Strand Buffer, 0.1 M DTT and RNase-free water. An incubation at 37 °C for 2 min and addition of M-MLV RT enzyme was performed next. The resulting solution was placed at 37 °C for 50 min and at 70 °C for 15 min. The synthesized cDNA was stored at 4 °C.

### 2.4. qRT-PCR Amplification of Transcripts of Interest

The primers for the amplification of Argonaute-1 (*Ago1*; LOC111515958), Argonaute-2a (*Ago2*; LOC111513702), Argonaute-3 (*Ago3*; LOC111510264), Dicer-2a (*Dcr2a*; LOC111517750), Dicer-2b (*Dcr2b*; LOC111509308), Exportin-5 (*Expo-5*; LOC111501848), Siwi-1 (LOC111515421) and Siwi-2 (LOC111505514) targets in *L. decemlineata* were designed using the Primer3Plus tool and have been reported previously in the literature [22]. Each primer pair amplified a PCR product of approximately 215 base pairs. The reagent mixture was as follows: 5 µL of 10-fold-diluted cDNA, 1 µL of 25 µM forward and reverse primers, 5.5 µL of RNase-free water and 12.5 µL of 2X Taq FroggaMix (FroggaBio, Concord, ON, Canada). The ensuing PCR amplification included a denaturing step of 95 °C for 5 min and 35 cycles of 95 °C for 15 s at a temperature gradient between 54 and 65 °C for 60 s and 72 °C for 45 s. Product identity was confirmed following the amplification by agarose gel electrophoresis and subsequent sequencing with the Université Laval sequencing platform (Quebec City, QC, Canada). Primer annealing temperatures and amplification efficiencies were assessed using qRT-PCR. Primer efficiencies were determined via amplification of the target in serial dilutions of cDNA followed by the measurement of the slope of the Cq of amplified target versus the quantity [23]. qRT-PCR-based quantification of the target transcripts in insects treated with cyantraniliprole or thiametoxam was performed in technical triplicates. A mixture of 2.5 µL of diluted cDNA (10^−1^), 0.5 µL RNase-free water, 1 µL 5 µM forward primer, 1 µL 5 µM reverse primer and 5 µL iTaq Universal SYBR Green Supermix (Bio-Rad, Hercules, CA, USA) was prepared. This mixture was subjected to a denaturing step at 95 °C for 3 min, 40 cycles at 95 °C for 15 s and a select hybridization temperature for 30 s. Transcript expression of *RP-18*, leveraged as a reference, was also assessed in parallel.

### 2.5. dsRNA Synthesis

*Ago1* was targeted using a dsRNA-based approach. The MEGAscript RNAi Kit (Thermo Fisher Scientific) was used to design dsRNA complementary to the *L. decemlineata Ago1* sequence following the manufacturer’s directions. The sequences for T7 primers used to generate dsRNA aimed at *Ago1* were forward 5′-TAATACGACTCACTATAGGGAGAACCAGTATTCGATGGCAGGA-3′ and reverse 5′-TAATACGACTCACTATAGGGAGACGGAAATGACTGCATCTGTG-3′. Amplification was initially conducted using a PCR-based approach performed at 95 °C for 5 min, 39 cycles at 95 °C for 15 s, 60 °C for 30 s and 72 °C for 45 s. QIAquick PCR Purification (QIAGEN, Hilden, Germany) was conducted to purify the amplified products. Sequencing of PCR products was conducted as before to confirm identity and prior to their utilization as a template for the synthesis of dsRNA. DNase and RNase treatments were performed on the amplified dsRNA, which was further quantified using a spectrophotometer. Synthesized dsRNA was stored at −20 °C until injection of insects.

### 2.6. dsRNA Injection

Injection of insects was performed with a 10 µL syringe (Hamilton, Reno, NV, USA). Adult insects were immobilized on their backs while conducting the dsRNA injection. Insect abdomens were injected with a volume corresponding to 5 µL of a 482.2 ng/µL *Ago1*-targeting dsRNA. A similar volume of the saline solution in which dsRNA was diluted was injected into the abdomens of control insects. Insects were maintained in an incubator under the settings described above following dsRNA injection.

### 2.7. Ago1 Silencing Analysis

qRT-PCR was conducted to measure *Ago1* levels in injected insects one week following dsRNA injection. TRIzol was used to obtain total RNA isolates that were each generated using one insect. Primers used to assess target transcripts expression were designed near the 5′ extremity of the target and outside the dsRNA-targeted region. Transcript levels were measured via amplification at 95 °C for 3 min, 40 cycles at 95 °C for 15 s and a hybridization temperature for 30 s. Transcript levels of *RP-18* were also amplified in the investigated samples and used to normalize target expression. An unpaired Student’s *t*-test was used to evaluate significant differences in target expression between samples.

### 2.8. Insecticide Exposure Response in dsRNA-Injected L. decemlineata

Adult insects injected with dsRNA were subsequently treated with cyantraniliprole to evaluate the potential impact of *Ago1* variation on the susceptibility against this compound. The effect of cyantraniliprole was investigated by initially separating the insects into two different groups (*n* = 32) in Petri dishes that included control insects as well as insects injected with *Ago1*-targeting dsRNA. Topical application was conducted ten days following injection by pipetting onto the abdomens of select insects (*n* = 11–13) from each group a volume of 1 µL of a 0.05 µg/µL cyantraniliprole solution in acetone, yielding a final dose of 0.05 µg. The remaining insects (*n* = 11–13) from each group received an equal volume of acetone and served as controls. Insect impairment was assessed as described above over the next four weeks following treatments with cyantraniliprole.

### 2.9. Quantification and Statistical Analysis

Quantification cycle data and relative normalized transcript expressions were collected and processed using CFX Manager v3.1 and CFX Maestro v4.1 (Bio-Rad). Unique peaks following qRT-PCR runs were observed with melt curve analysis. Box and whisker plots were used to identify outliers. Statistical significance of transcript expression in treated versus control insects was evaluated using an unpaired Student’s *t*-test with GraphPad Prism v9.5.0. A log-rank test was also conducted to assess the differences observed in survival curves using the same software.

## 3. Results

### 3.1. Quantification of Transcripts Associated with Small Non-Coding RNA Processing in L. decemlineata Treated with Cyantraniliprole

**Figure 1:**
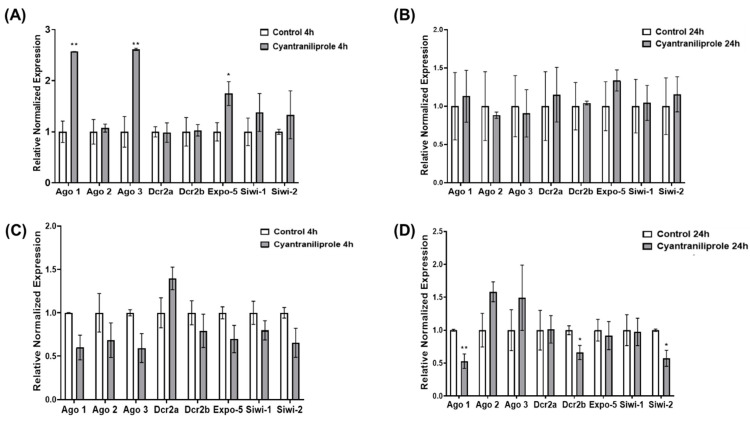
Transcript levels measured via qRT-PCR in *L. decemlineata* exposed to cyantraniliprole. Data present mean standardized transcript levels (mean ± SEM, *n* = 3–5 biological replicates). Histograms depict levels of transcripts in insects exposed to low doses of cyantraniliprole for 4 h (**A**) or 24 h (**B**). Histograms present transcript levels for the same targets in insects treated with high doses of cyantraniliprole for 4 h (**C**) or 24 h (**D**). Asterisks identify results that differ significantly from controls (* *p* < 0.05 and ** *p* < 0.01).

Adult *L. decemlineata* were treated with low and high doses of cyantraniliprole for different time durations. The transcript levels of *Ago1*, *Ago2*, *Ago3*, *Dcr2a*, *Dcr2b*, *Expo-5*, *Siwi-1* and *Siwi-2* were subsequently assessed via qRT-PCR. *Ago1* transcript expression showed an up-regulation of 2.57-fold (*p* < 0.01) in insects that were treated with low doses of cyantraniliprole for a period of four hours versus insects used as controls. *Ago3* (*p* < 0.01) and *Expo-5* (*p* < 0.05) expression also displayed increases in insects subjected to the same conditions (Figure 1A). The transcript levels of the eight targets assessed did not display substantial variations in insects exposed to a low dose of cyantraniliprole for 24 h (Figure 1B). No statistically significant difference was recorded in insects that were treated with a high dose of cyantraniliprole for a duration of four hours (Figure 1C). On the other hand, reduced transcript levels were observed following high-dose cyantraniliprole exposure for a period of 24 h (Figure 1D). *Ago1* transcript levels displayed reduced expression in cyantraniliprole-exposed insects to levels that were 0.53-fold (*p* < 0.01) higher than the ones recorded in the controls. Reduced levels of transcripts coding for *Dcr2b* and *Siwi-2* were also observed in treated insects to levels, respectively, 0.66-fold and 0.57-fold higher than the ones measured in the control insects (*p* < 0.05).

### 3.2. Quantification of Transcripts Associated with Small Non-Coding RNA Processing in L. decemlineata Treated with Thiamethoxam

**Figure 2:**
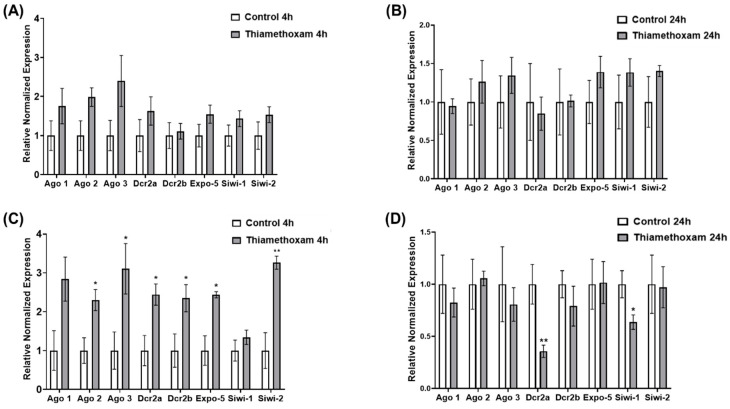
Expression of transcript levels as determined via qRT-PCR in *L. decemlineata* treated with thiamethoxam. Results shown are mean standardized transcript levels (mean ± SEM, *n* = 3–5 biological replicates). Data presented represent expression of small non-coding RNA transcripts in insects treated with a low dose of thiamethoxam for a period of 4 h (**A**) or 24 h (**B**). Histograms display the expression status of the same targets of interest in insects exposed to thiamethoxam for 4 h (**C**) or 24 h (**D**). Asterisks represent results that are significantly different (* *p* < 0.05 and ** *p* < 0.01).

The expression levels of the eight investigated targets were also monitored in adult *L. decemlineata* treated with thiamethoxam for similar periods of time. Transcripts coding for *Ago1*, *Ago2* and *Ago3* were all over-expressed, albeit not significantly, following a low-dose thiamethoxam treatment for four hours with changes of 1.75-fold, 1.98-fold and 2.40-fold in insecticide-treated versus control insects, respectively (Figure 2A). Insect exposure to a similar dose of thiamethoxam for a period of 24 h was not associated with substantial changes in the expression of the investigated transcripts (Figure 2B). Significant increases were seen in insects exposed to a high dose of thiamethoxam for four hours, with changes observed for several transcripts including *Ago2*, *Ago3, Dcr2a*, *Dcr2b* and *Expo-5* (*p* < 0.05) (Figure 2C). This treatment also resulted in the significant over-expression of *Siwi-2* transcript levels with variations of 3.26-fold (*p* < 0.01) in insecticide-treated insects. Changes in expression status were observed in insects treated with high doses of thiamethoxam for a period of 24 h. This condition was associated with reduced *Dcr2a* and *Siwi-1* expression, with values in thiamethoxam-exposed insects that were 0.36-fold (*p* < 0.01) and 0.64-fold (*p* < 0.05) higher than the levels measured in the controls, respectively (Figure 2D).

### 3.3. L. decemlineata Mortality in Cyantraniliprole-Exposed Insects following dsRNA Injection

**Figure 3:**
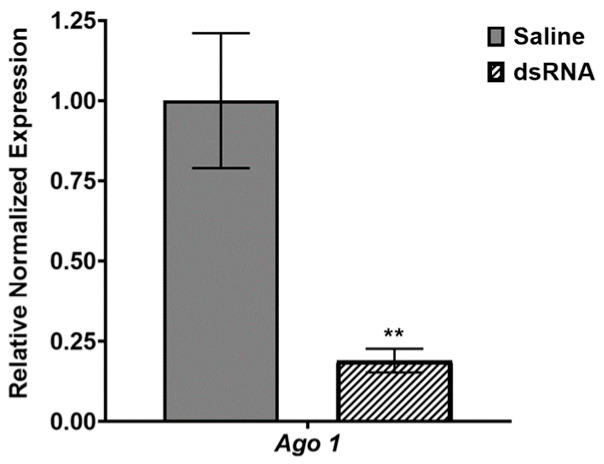
Quantification via qRT-PCR of *Ago1* transcript levels in *L. decemlineata* following dsRNA injection. Figure depicts levels of the target of interest in insects injected with dsRNA or insects injected with a saline solution. Results represent standardized transcript levels (mean ± SEM, *n* = 4). Transcript levels measured in dsRNA-injected insects that are significantly different from the levels observed in control insects are represented by asterisks (** *p* < 0.01).

Several transcripts of interest displayed changes in expression, as measured by qRT-PCR, following insecticide treatments in *L. decemlineata*. The status of transcripts coding for targets such as *Ago2* and *Ago3* displayed substantial up-regulation in insects following cyantraniliprole or thiamethoxam treatments for select time durations. The transcript levels of *Ago1* were notably up-regulated post-treatment in insects exposed, for four hours, to cyantraniliprole under a low-dose regimen. An RNAi-based approach was thus undertaken to assess the role, if any, of this target in cyantraniliprole susceptibility in *L. decemlineata*. *Ago1* modulation was successfully conducted with dsRNA via injection. The levels of transcripts coding for *Ago1* in dsRNA-injected insects were reduced to 0.19-fold (*p* < 0.01) of the levels measured in controls (Figure 3).

**Figure 4:**
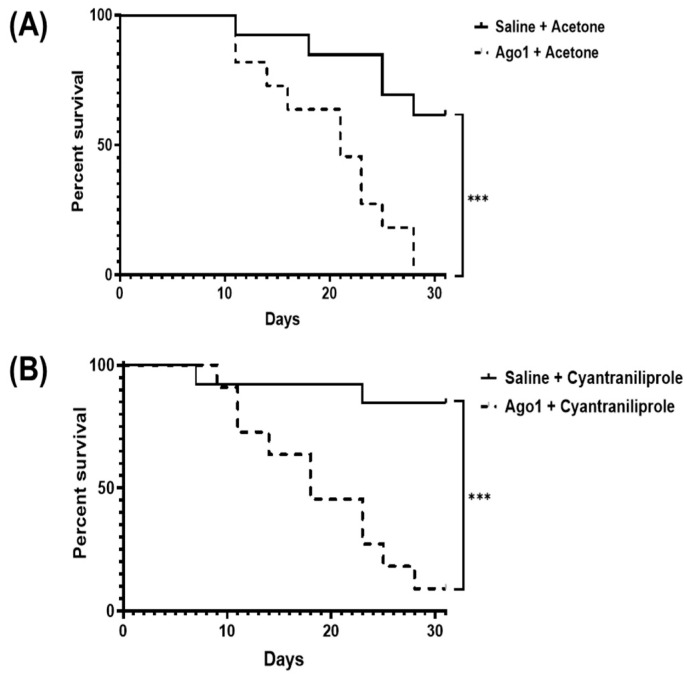
Impact of cyantraniliprole exposure in *L. decemlineata* injected with saline or *Ago1*-targeting dsRNA. Analysis presents insect viability in control versus dsRNA-*Ago1*-injected insects treated with 0.05 µg of cyantraniliprole. (**A**) Viability curve representing the effect of *Ago1* variation in insects exposed to acetone. (**B**) Survival percentage showing the impact of *Ago1* modulation on cyantraniliprole-exposed insects. Asterisks identify results that differ significantly (*** *p* < 0.001).

Insect viability was then assessed in the control or dsRNA-injected *L. decemlineata* exposed to cyantraniliprole. Changes in insect mortality were observed between saline-injected insects versus dsRNA-*Ago1*-injected insects subjected to acetone treatment. Insect survival was 61.5% in control insects, while no activity was detected in *Ago1*-injected insects four weeks following acetone exposure (Log-rank test *p* = 0.0008) (Figure 4A). A similar trend was observed in insects that were injected with dsRNA targeting *Ago1* or used as controls and subsequently exposed to cyantraniliprole. Insect survival was 84.6% in saline-injected insects exposed to cyantraniliprole, while survival was 9.1% in *Ago1*-injected insects four weeks following cyantraniliprole treatment (Log-rank test *p* = 0.0003) (Figure 4B).

## 4. Discussion

A growing number of studies have presented variations in small non-coding RNA expression in a variety of insects as a result of treatments with different chemical compounds or have linked such modulation with confirmed resistance to a given insecticide. On the other hand, information is sparse regarding the levels of transcripts coding for proteins underlying pathways associated with small non-coding RNA processing in insects treated with or resistant to a given chemical. This study was conducted to evaluate the expression status of select transcripts involved in such pathways in *L. decemlineata* following treatments with varying doses of the diamide cyantraniliprole or the neonicotinoid thiamethoxam. Information gathered here demonstrated signatures of transcript expression that were different based on the dose or length of the treatments conducted.

Various targets involved in the response to diamide exposure have been explored and identified in multiple insects. Pioneering work conducted on the Asian corn borer, *Ostrinia furnacalis*, revealed several up-regulated genes, including certain genes coding for cytochrome P450 monoxygenases, following treatment with flubendiamide [24]. A subsequent study performed on the rice stem borer, *Chilo suppressalis*, demonstrated the modulation of multiple targets, including several linked to xenobiotic metabolism in larvae exposed to the diamide chlorantraniliprole [25]. Different reports have also shown the modulation of small non-coding RNAs following exposure to diamide insecticides in several insects. The miRNAs *miR-7a* and *miR-8519* have been shown to regulate the expression of *PxRyR*, a key target associated with chlorantraniliprole susceptibility in the diamondback moth, *Plutella xylostella* [26]. Expression profiling of miRNAs in fall armyworms, *Spodoptera frugiperda*, subjected to tetraniliprole was associated with varied levels of 30 miRNAs, and subsequent *miR-278-5p* modulation could impact the response to this compound in this agricultural pest [27]. Results from the current study highlight a modulation of *Ago1* transcript levels in adult *L. decemlineata* exposed to the diamide cyantraniliprole. A marked up-regulation of *Ago1* transcripts was observed in insects exposed to a low dose of this compound for a short duration. Interestingly, treatment with a high dose of cyantraniliprole for a longer period was associated with the down-regulation of *Ago1* and *Dcr2b* transcript levels. This aligned with recent work that showed a reduction in select transcripts associated with small non-coding RNA processing, including *Ago1* and *Dcr2*, in cotton bollworms, *Helicoverpa armigera*, fed a diet containing fenoxycarb for periods of 24 h or longer [28]. While subsequent work is envisioned to further explore the impact of cyantraniliprole on the expression of genes with potential relevance to small non-coding RNAs, the results gathered here notably present the differential expression of *Ago1* following exposure to this diamide in *L. decemlineata*.

Much information is available regarding the potential role played by small non-coding RNAs in the response and resistance of insects against neonicotinoid insecticides such as thiamethoxam. Pioneering work on this topic has highlighted the modulation of 15 miRNAs in honey bee larvae that had been administered imidacloprid-containing syrup, showcasing a miRNA response mediated by this neonicotinoid [29]. Deep sequencing of small RNAs in whiteflies, *Bemisia tabaci*, exposed to imidacloprid revealed the differential expression of 16 miRNAs in a strain resistant to this compound when compared with a susceptible counterpart [30]. Another study demonstrated that varying the expression levels of three miRNAs could influence imidacloprid responses in the grain aphid, *Sitobion miscanthi* [31]. Examples in the literature highlighting a miRNA response following insect treatment with thiamethoxam specifically are sparse. Nevertheless, exposing honey bees to a thiamethoxam dose deemed comparable to levels encountered in the field was associated with the significant differential expression of seven miRNAs [13]. The data presented here show a significant down-regulation of *Dcr2a* and *Siwi-1* transcript levels in *L. decemlineata* exposed to a high dose of thiamethoxam for 24 h. A significant reduction in these transcripts was also observed in this insect when it was subjected to other neonicotinoids, such as imidacloprid for the former and clothianidin for the latter [22], strengthening the potential implication of these targets in *L. decemlineata*’s response to this family of compounds. This information adds to a growing body of knowledge that supports the modulation of small-non-coding-RNA-associated targets in *L. decemlineata* treated with thiamethoxam. It is nevertheless important to point out that further exploration of specific causes underlying the observed differences in the expression of transcripts resulting from either cyantraniliprole or thiamethoxam exposure in *L. decemlineata* is warranted to better understand the compound-specific variations reported in this work. This notably includes revisiting the overall mechanisms of action associated with the studied compounds [32,33] and their possible effects on insecticide responses. Assessing transcript expression in *L. decemlineata* exposed to additional compounds that are either related to cyantraniliprole or thiamethoxam would also help to evaluate whether the changes reported here could be part of a larger response against diamide or neonicotinoid insecticides, respectively.

The changes in *Ago1* expression status following cyantraniliprole exposure in *L. decemlineata* reported above warranted the further exploration of the role of this transcript, if any, in the response of this insect pest toward this chemical. It is noteworthy to add that *Ago1* was the only transcript target investigated in this work that displayed fluctuating levels following the different cyantraniliprole treatment conditions assessed, further supporting its selection for subsequent dsRNA-based investigations. The efficient knockdown of *Ago1* transcript levels using dsRNA-based approaches has been reported elsewhere in various insects, including the western corn rootworm, *Diabrotica virgifera virgifera* [34], and the oriental fruit fly, *Bactrocera dorsalis* [35]. Furthermore, the dsRNA-mediated down-regulation of the expression of three transcripts coding for chemosensory proteins in a cyantraniliprole-resistant strain of the cotton aphid, *Aphis gossypii*, is associated with increased susceptibility toward this compound [36]. The current study presents a successful reduction in *Ago1* transcript levels in adult *L. decemlineata* using dsRNA. In addition, the results collected here show that this down-regulation had a positive impact on cyantraniliprole susceptibility in this potato pest. The knockdown of *Ago1* levels in dsRNA-injected insects, without subsequent cyantraniliprole treatment, also highlighted the impact of *Ago1* on insect viability when compared with the saline-injected insects used as controls. *Ago1*, thus, joins other examples of molecular leads that warrant further studies to evaluate their potential involvement in cyantraniliprole susceptibility in insects, including the inositol 1,4,5-trisphosphate receptor (*IP3R*) in the whitefly, *B. tabaci* [37], or *CYP6DA1* in *A. gossypii* [38]. Subsequent explorations of the dsRNA-based modulation of additional transcripts coding for Argonaute proteins—such as *Ago1* or *Ago2*—and transcripts—including *Dcr2a* and *Siwi-2*—that are overexpressed in response to thiamethoxam are also warranted. This would contribute to a better understanding of their potential role in *L. decemlineata*’s response to this neonicotinoid. These up-regulated transcripts could ultimately be used to develop RNAi-based strategies against *L. decemlineata* and aimed at novel molecular targets, as conducted successfully by other groups on genes such as *Mesh* [39] and *PSMB5* [40], to name a few. Overall, the collected dsRNA-based results show the impact of modulating *Ago1* transcript levels in *L. decemlineata* either exposed or not to the diamide cyantraniliprole and warrant further investigation of this transcript target in this potato pest.

## 5. Conclusions

The current work reports the differential expression of multiple transcripts coding for proteins that have functions associated with small non-coding RNA processing in *L. decemlineata* insects treated with cyantraniliprole or thiamethoxam. Significant variation in *Ago1* transcript expression was observed in insects following a low-dose treatment of cyantraniliprole for a short duration and a high dose of the same compound for a longer period. The treatment of insects with a high dose of thiamethoxam for a prolonged duration was linked to changes in the expression status of transcripts coding for *Dcr2a* and *Siwi-1*. *Ago1* transcript levels were also shown to be modulated, albeit not significantly, in insects exposed to low and high doses of thiamethoxam for a short duration. A dsRNA-based approach was used to investigate the role of *Ago1* in cyantraniliprole responses. A significant elevation in mortality was observed in insects injected with dsRNA directed at *Ago1*, supporting its potential role in cyantraniliprole responses in *L. decemlineata*. Follow-up experiments are envisioned to further build on the information gathered. These include the assessment of changes, if any, in small non-coding RNA levels in *L. decemlineata* treated with the compounds of interest, as well as the analysis of the impact of modulating select overexpressed transcripts following thiamethoxam exposure, such as *Ago2* and *Ago3*, on *L. decemlineata*’s response to this compound. The investigation of possible mutations in *L. decemlineata* populations with confirmed resistance against cyantraniliprole or thiamethoxam should also be considered to identify mutations in specific genes of interest that could mediate insecticide resistance. Overall, this work reported transcripts associated with small non-coding RNA processing pathways that showed changes in expression following cyantraniliprole and thiamethoxam exposure in *L. decemlineata* and supports additional studies to better assess the role, if any, of such pathways with respect to insecticide response in agricultural pests.

## Data Availability

The data presented in this study are available from the corresponding authors upon request.

## Supplementary Materials

The following are available online at https://www.mdpi.com/article/10.3390/insects15030147/s1: Table S1: Effect of cyantraniliprole or thiamethoxam treatments on adult *L. decemlineata*.
