# Supplementary material for: Cyantraniliprole and Thiamethoxam Exposure Changes Expression of Transcripts Associated with Small Non-Coding RNA Processing in the Colorado Potato Beetle
Source: Insects. 2024 Feb 22;15(3):147. doi: 10.3390/insects15030147 (PMC10971089; doi:10.3390/insects15030147)
Supplement: Supplementary file 1 [file insects-15-00147-s001.zip › insects-2841335-supplementary.pdf]

Table S1

(A)

Dose	Insects (impaired/total)	Mortality
Control	0/20	0 %
0.01 µg	1/20	5 %
0.05 µg	4/20	20 %
0.1 µg	10/20	50 %
0.5 µg	16/20	80 %
1.0 µg	18/20	90 %
5.0 µg	19/20	95 %

(B)

Dose	Insects (impaired/total)	Mortality
Control	1/20	5 %
0.01 µg	1/20	5 %
0.05 µg	4/20	20 %
0.1 µg	4/20	20 %
0.5 µg	14/20	70 %
1.0 µg	17/20	85 %
5.0 µg	19/20	95 %

Table S1. Effect of cyantraniliprole or thiamethoxam treatments on adult *L. decemlineata*. Table shows impact on insects treated to different doses of these compounds for a period of 24 hours. Results presented for *L. decemlineata* exposed to (A) cyantraniliprole or (B) thiamethoxam. Control insects were treated with acetone.
